# Electrodiagnostic studies in presumptive primary hypothyroidism and polyneuropathy in dogs with reevaluation during hormone replacement therapy

**DOI:** 10.1186/s13028-016-0212-9

**Published:** 2016-05-21

**Authors:** Elżbieta Gabriela Giza, Marta Płonek, Józef Marian Nicpoń, Marcin Adam Wrzosek

**Affiliations:** Department of Internal Medicine and Clinic of Horses, Dogs and Cats, Faculty of Veterinary Medicine, Wroclaw University of Environmental and Life Sciences, pl. Grunwaldzki 47, 50-366 Wroclaw, Poland

**Keywords:** Electrodiagnostic, BAER, Levothyroxine, Hypothyroidism, Polyneuropathy, Dog

## Abstract

**Background:**

Peripheral neuropathy is the most common neurological manifestation of canine hypothyroidism. Data concerning electrodiagnostic studies in hypothyroid associated polyneuropathy in dogs are very limited and usually lack a reevaluation after hormone replacement therapy. The objective of this study was to perform a detailed, retrospective analysis of electromyographic (EMG), motor nerve conduction velocity (MNCV), F-wave and brainstem auditory evoked response (BAER) findings in 24 dogs with presumptive primary hypothyroidism and polyneuropathy with a comparison of the results before and after initiation of levothyroxine treatment with the assessment of the clinical outcome.

**Results:**

The results obtained from hypothyroid dogs showed a significant reduction in MNCV at a proximal–distal and middle–distal stimulation, decreased amplitudes of compound muscle action potentials (CMAP), an increased CMAP duration and a prolonged distal latency prior to treatment. Fifty percent of the dogs had an increased F-wave latency. A normal BAER recording was found in 78 % of the hypothyroid patients without vestibular impairment. Bilaterally increased peak V latencies and increased interpeak I–V latencies were found in the remaining individuals. Dogs with concurrent vestibular impairment had ipsilaterally increased peak latencies with normal interpeak latencies and decreased amplitudes of wave I and II. A comparison of the findings before and after 2 months of treatment revealed a decrease in the pathological activity on EMG, an improvement of proximal, middle and distal CMAP amplitudes and an increase in the proximal–distal conduction velocity in all dogs. F-wave latency improved in 38 % of dogs. The BAER reexamination revealed a persistent prolongation of peak I, II, III and V latencies and decreased wave I amplitude on the affected side in all dogs manifesting vestibular signs. Conversely, in dogs without vestibular signs, the peak V and interpeak I–V latencies decreased to normal values after a given time of the treatment.

**Conclusions:**

The results indicate a demyelinating and axonal pattern of polyneuropathy in dogs with suspected hypothyroidism. Most of the patients without vestibular signs showed neither peripheral nor central auditory pathway impairment, concurrent to the generalized neuropathy. The follow-up examination showed a very good clinical outcome and only partial improvement in electrophysiological assessment.

## Background

Canine hypothyroidism is the most common endocrinopathy in mature, middle- to large-sized dogs and is characterized by a range of clinical manifestations [1, 2]. While the classical signs of this disease are well recognized, the correlation between hypothyroidism and neurologic dysfunction is less known [3]. Both central and peripheral neurological signs have been described in veterinary medicine [4]. Although the brain is considered relatively resistant to hypothyroidism in animals [5], seizures, disorientation, circling, coma and brainstem abnormalities, including central vestibular signs have been reported in hypothyroid dogs [3, 6, 7]. Peripheral nervous system syndromes, including cranial neuropathies (trigeminal, facial and vestibulocochlear nerve dysfunction), laryngeal paralysis, megaesophagus and, most frequently, generalized neuromuscular signs, have been reported in canine hypothyroidism [4, 7, 8].

The pathophysiology of polyneuropathy in hypothyroidism is not well understood [9]. Based on human studies, it is hypothesized that decreased metabolism in neurons may lead to impaired axonal transport and, in consequence, axonal atrophy [10, 11]. Another explanation for polyneuropathy is demyelination that is caused by the deposition of mucopolysaccharides in the cytoplasm of Schwann cells and connective tissue of the nerve [9, 12]. It may also be a result of vascular damage due to disturbances in the blood-nerve barrier [13, 14].

Polyneuropathy and myopathy associated with thyroid hormone deficiency in dogs may present as exercise intolerance, fatigue, a stiff gait, muscle atrophy, intermittent lameness, para- and tetraparesis [15–17]. Hypothyroid animals may have decreased proprioception, hypotonia and decreased to absent spinal reflexes [15]. According to previous reports, the clinical signs of peripheral nerve dysfunction and central vestibular disease in hypothyroid dogs typically resolve after 2 months of levothyroxine supplementation [6, 18, 19].

The electrodiagnostic findings in canine hypothyroidism with neurological manifestations include increased insertional activity and abnormal spontaneous activity during electromyography (EMG), reduction in the amplitudes of compound muscle action potentials (CMAP), a decreased motor nerve conduction velocity (MNCV) and brainstem auditory evoked response (BAER) abnormalities [6, 8, 9]. Most of these studies were carried out on a very small number of dogs (case reports and case series). A single electrodiagnostic test without reevaluation after levothyroxine supplementation was performed in the majority of cases, particularly if complete resolution of the clinical signs was achieved.

The purpose of this study was a retrospective evaluation of EMG, MNCV, F-wave and BAER findings in 24 dogs with presumptive naturally occurring hypothyroidism and peripheral neuropathy, and a comparison of the results before and after initiation of levothyroxine supplementation with the assessment of the clinical outcome.

## Methods

### Case selection

Electrodiagnostic recordings from 34 dogs referred for a neurological consultation to the Department of Internal Medicine with the Clinic of Horses, Dogs and Cats, Wroclaw University of Environmental and Life Sciences between February 2010 and February 2014, were analyzed retrospectively.

Group A consisted of 24 dogs with a presumptive diagnosis of hypothyroidism and polyneuropathy based on the following criteria: (1) a hypothyroid state with a high probability of primary hypothyroidism based on clinical findings, the hemato-biochemical blood profile and thyroid function test (decreased T4, fT4 and increased cTSH; if cTSH normal—the presence of other laboratory abnormalities and at least two extraneural clinical signs indicative of hypothyroidism), (2) neurological signs of progressive, generalized lower motor neuron impairment, (3) an electrodiagnostic evaluation, confirming peripheral neuropathy, (4) a positive clinical response to thyroid hormone replacement therapy (THRT), (5) an unremarkable abdominal ultrasound, chest x-rays, cerebrospinal fluid examination, (6) a normal head MRI in cases with cranial nerves deficits. In order to be included in the study, patients could not have a history of recent trauma or surgery, exposure to toxins or treatment with phenobarbital.

The age of the examined animals at presentation ranged between 5 and 12 years (average: 9 years, median 8.5 years) and included 9 males (1/9 neutered) and 15 females (11/15 spayed). Subgroup A1 consisted of 11 dogs that underwent an electrodiagnostic reexamination 2 months after starting THRT. The detailed data regarding the breeds of dogs, history and electrodiagnostic tests is given in Table 1.

**Table 1:** Results of the clinical and electrodiagnostic evaluations in individual hypothyroid dogs before and after treatment

Dog No.	Breed	Age [years] sex	Motor deficits BT, other signs	Motor deficits AT, other signs	EMG		MNCV[m/s]				Expected F-wave lat.[ms]	F-wave lat.[ms] BT	F-wave lat.[ms] AT
					BT	AT	Proximal–distal		Middle-distal				
							BT	AT	BT	AT			
1	Bernese M. Dog	8 ♀	3	1	4	3	26	28	20	24	21.6	24.1	24
2	Golden Retriever	7 ♂	2, Vestib.	1	3	2	31	42	30	31	20.6	26.8	22
3	Golden Retriever	11 ♀	2	1	3	2	32	34	28	28	18.6	21.3	21.1
4	Labrador Retriever	6 ♀	3, Vestib.	1	4	3	35	44	31	34	19.6	32.2	30.1
5	Labrador Retriever	11 ♀	2	1	4	2	35	38	33	35	20.3	29.3	29.4
6	Irish Setter	5 ♀	2	1	3	2	39	40	36	36	21.3	29.5	24.7
7	Flat-coat. Retriever	12 ♀	2	1	3	2	41	48	36	39	19.6	20.2	NP
8	Mongrel (35 kg)	11 ♂	3	1	4	2	44	46	39	41	19.0	20.1	NP
9	Mongrel (15 kg)	10 ♀	3	1	3	1	44	48	41	42	16	16.5	NP
10	Wire Fox Terrier	12 ♂	2	1	3	1	46	55	41	44	15.1	18.9	16.5
11	WHWT	11 ♀	3	1	3	1	47	58	45	46	12.7	16.2	15.9
12	Labrador Retriever	9 ♂	2	0	3	NP	45	NP	42	NP	NP	NP	NP
13	Irish Setter	11 ♂	2	0	3	NP	45	NP	39	NP	21.6	23	NP
14	German Shepherd	7 ♂	2, Vestib.	0	3	NP	46	NP	43	NP	NP	NP	NP
15	Golden Retriever	8 ♀	2	0	3	NP	48	NP	47	NP	18.6	20.4	NP
16	Eng. Cocker Spaniel	6 ♀	2, Vestib.	0	3	NP	48	NP	44	NP	14.7	15	NP
17	Golden Retriever	7 ♂	1	0	3	NP	48	NP	43	NP	NP	NP	NP
18	Mongrel (20 kg)	8 ♀	1, Vestib.	0	3	NP	49	NP	47	NP	NP	NP	NP
19	Min. Schnauzer	11 ♂	1	0	3	NP	50	NP	45	NP	NP	NP	NP
20	Min. Poodle	12 ♀	1	0	3	NP	50	NP	44	NP	NP	NP	NP
21	Dachshund	8 ♀	1	0	2	NP	52	NP	48	NP	11	11.9	NP
22	Golden Retriever	6 ♀	1	0	2	NP	53	NP	46	NP	18.6	18.5	NP
23	Golden Retriever	9 ♂	1	0	2	NP	53	NP	48	NP	NP	NP	NP
24	Maltese	10 ♀	1	0	2	NP	54	NP	47	NP	NP	NP	NP

Dogs are ranked in ascending order, according to proximal–distal MNCV before the treatment. The ranking is separate for cases No. 1–11 (group A1) and for the remaining 13 cases

*NP* not performed, *BT* before treatment, *AT* after treatment [2 months], *WHWT* west highland white terrier

Group B (control group) consisted of ten dogs that were initially admitted for a neurological examination, but were diagnosed with an orthopedic problem and were otherwise healthy. The age of the dogs was similar to those from group A, and ranged between 5 and 10 years (average 8 years, mean 7 years). This group consisted of 4 males and 6 females (4/6 spayed). The body weight ranged from 11 to 46 kg (mean 23 kg, median 26 kg). Hypothyroidism was excluded in this group based on normal blood test results. MNCV recordings, CMAP latencies, amplitudes and durations as well as BAER test recordings were documented. All the results were considered normal for the given age, size and limb length of the dogs based on a comparison with a previous report [20].

### Clinical examination

All dogs were examined clinically, orthopedically, and neurologically. Motor function was evaluated and motor deficits were classified according to the following scale: Grade 0—normal motor function and muscle strength, Grade 1—mild motor deficits, muscle weakness, ambulatory paresis; Grade 2–moderate deficits, non-ambulatory paresis and Grade 3—severe deficits, complete loss of strength, plegia. A basic laboratory workup for neuromuscular disease according to Cuddon [9], including a hemato-biochemical blood profile and urinalysis was performed in all dogs. The thyroid function tests included measurements of total thyroxine (T4) (normal range 1.4–4.5 μg/dl), free thyroxine (fT4) by equilibrium dialysis (normal range 8–40 pmol/L), and serum canine thyroid stimulating hormone (cTSH) concentrations (normal range 0.01–0.50 ng/mL).

The electrophysiological examination was carried out at an ambient temperature of 22 °C with the use of the Nicolet Viasys Healthcare portable electrodiagnostic equipment and the version 11.0 of the Viking Quest software. Prior to testing, dogs were premedicated with a combination of medetomidine at a dose of 20 μg/kg and butorfanol at a dose of 0.1 mg/kg administered in the triceps brachii muscle. The electrodiagnostic examination was performed under general anesthesia using propofol at an induction dose of 1–4 mg/kg and a maintenance dose of 0.1–0.4 mg/kg/min, respectively.

The EMG was performed in all dogs in both groups by intramuscular insertions of a standard concentric needle electrode into the appendicular and axial muscles, with the monopolar ground electrode placed subcutaneously on the animal’s flank. A semi-quantitative numerical scale by Kimura [21] was modified and adopted for the purpose of this study to evaluate the severity of EMG changes (Table 2).

**Table 2:** Modified semi-quantitative numerical scale for the electromyographic evaluation of the degree of pathological potentials

Grade	Description
0	No pathological potentials
1	Very rare denervation potentials
2	Sporadic pathological activity recorded in two or more places
3	Frequent pathological activity recorded regardless of the position of the needle electrode
4	Abundant pathological activity recorded regardless of the position of the needle electrode

The MNCV study and F-wave assessment were performed with the use of: (1) two stimulating needle electrodes, (2) a concentric recording needle electrode, placed in the plantar interosseous muscle and (3) a ground electrode, placed between the stimulating and recording electrodes. The MNCV measurements were obtained in dogs in both groups in the sciatic/tibial nerves using a single supramaximal electrical stimulus with a 0.1 ms (ms) duration at three stimulation points according to Walker et al. [22]: proximal—caudal to the greater trochanter of the femur; middle—at the level of stifle and caudal to the distal end of the femur; and distal—above the hock, lateral to the gastrocnemius tendon of the tuber calcanei. The recordings of the MNCV and CMAP latency, duration and amplitude were analyzed and compared between groups A and B. The same variables were analyzed in group A1, before and 2 months after initiating THRT. The F-wave evaluation was based on the measurement of the latency of the recorded F-waves in comparison to the expected minimum F-wave latency calculated according to the following formula $$3.45\,+\, 0. 3 3\times{\text{limb length}}\,({\text{cm}})\; \text {for the sciatic/tibial {nerve}}$$ [20]. The limb length was measured on an extended limb from the level of the greater trochanter of the femur to the distal tip of the 3rd digit. Responses were elicited by 16 stimuli and the smallest latency period before the F-wave complex was measured.

The BAER test was performed in both groups of dogs using a previously described technique [20]. Briefly, standard subcutaneous needle-electrodes were placed in the mastoid reference (M), where the recording electrode was positioned at the vertex, the reference electrode was placed at the base of the ear and the ground electrode was placed at the midline of the 1st cervical vertebra. The auditory stimulus (1000 sweeps) was emitted through tubal insert ear phones at a 90 dB nHL intensity to the tested ear. A masking noise of 60 dB was simultaneously applied to the contralateral ear. All recordings were performed in rarefaction polarity, with a frequency setting of 11 Hz. The analysis of the BAER recording, obtained from the both ears in each individual, included an identification of waves I–V, measurement of their amplitudes and latencies and the determination of the I–V interpeak latency (IPL). All those variables were compared between the dogs in group A and B. In case of abnormal BAER recordings at the first examination, the comparison of the results before and after the initiation of the therapy was additionally performed in each individual. The BAER examination was preceded by an otoscopic examination.

The follow-up examination, which included a reevaluation of the clinical and neurological status as well as carrying out thyroid function tests was performed approximately 1 month after starting THRT at a dose of 20 µg/kg twice daily (BID) in all dogs in group A. The dosage was further adjusted according to the clinical response, the results of laboratory tests and clinical findings. Electrodiagnostic reevaluation was performed 2 months after initiating the THRT and was always conducted by this same examiner for the respective cases. Detailed information on the individual cases is presented in Table 1.

The results of the electrodiagnostic measurements were subjected to statistical analysis, using Excel 6.0 software and StatSoft Inc Statistica 10 software. The non-parametric Mann–Whitney U test was used to compare non-parametric values in the hypothyroid and control group. Student’s *t* test was used to compare parametric data before and after starting levothyroxine treatment. *P* < 0.05 was considered significant.

## Results

### Clinical and electrodiagnosting findings prior to levothyroxine supplementation

All of the 24 dogs in group A (hypothyroid) were presented with generalized weakness and hyporeflexia. Lethargy was noted in 16/24, excess weight in 14/24, bradycardia in 8/24 and typical dermatological signs in 5/24. Motor deficits were evaluated as Grade “1” in 8/24 dogs, Grade “2” in 11/24 individuals and Grade “3” in 5/24 canine patients (Table 1). Five hypothyroid patients additionally manifested signs of peripheral vestibular dysfunction (head tilt, horizontal nystagmus, vestibular strabismus). The duration of clinical signs varied from 1 to 12 weeks (median 6 weeks, mean 5 weeks) with a slow progression of generalized weakness in all dogs. According to the owners’ reports, the motor deficits were acutely exacerbated in seven individuals prior to the consultation. The onset of vestibular signs was also acute in all cases.

The diagnosis of hypothyroidism in group A was based on laboratory findings including decreased tT4 (24/24), decreased fT4 (22/24) and increased cTSH in 15/24. All dogs with normal cTSH (9/24) had moderate to severe fasting hypercholesterolemia, 7/9 dogs had fasting hypertriglyceridemia, 4/9 were presented with normocytic anemia, 2/9 had mildly increased aspartate aminotransferase and creatinine kinase activity. In total, 22/24 dogs in group A had hypercholesterolemia, 18/24 had hypertriglyceridemia, 7/24 had anemia and 6/24 dogs had a mildly increased aspartate aminotransferase and creatinine kinase activity.

The EMG examination in group A revealed increased insertional activity in 14/24 dogs, multifocal patterns of fibrillation potentials (FPs) in all 24 patients and positive sharp waves in 18/24 dogs. The changes were noticeable in all four limbs in all dogs in group A. The abnormal activity was equally appreciated in the proximal and distal parts of the front and hind limbs. Additional FPs were present in the axial muscles of eight dogs. The severity of individual EMG changes for each individual is shown in Table 1. The MNCV study in the hypothyroid group revealed a statistically significant reduction in the conduction velocity at the proximal–distal (26–54 m/s) and middle-distal stimulation (20–48 m/s) when compared to the control group (Table 3). The detailed analysis of CMAP parameters revealed a prolonged distal latency, decreased CMAP amplitudes and an increase in the CMAP duration at all stimulation points in group A (Table 3). The F-wave was recorded in 16 dogs from group A (Cases No. 1–11, 13, 15, 16, 21, 22). The F-wave latency was increased by more than 2 ms in eight patients (50 %) when compared to the expected F wave latency calculated for each dog (cases 1–6, 10, 11) (Table 1).

**Table 3:** Comparison of motor nerve conduction variables in hypothyroid dogs and healthy controls before and after treatment

Group parameter	Group A (hypothyroid) [n = 24]	Group B (control) [n = 10]	*P* value	Group A1 before THRT [n = 11]	Group A1 after THRT [n = 11]	*P* value
MNCV prox-dist [m/s]	44.2 ± 7.6	65.4 ± 2.5	<*0.05*	38.2 ± 6.9	45.7 ± 6.2	<*0.05*
MNCV mid-dist [m/s]	40.1 ± 7.3	60.8 ± 2.2	<*0.05*	34.5 ± 7.1	36.2 ± 6.3	0.57
Latency prox [ms]	9.2 ± 1.7	7.8 ± 0.4	0.31	–	–	–
Latency mid [ms]	7.1 ± 1.6	5.8 ± 0.3	0.25	–	–	–
Latency dist [ms]	6.1 ± 1.1	3.0 ± 0.4	<*0.05*	5.6 ± 1.1	5.5 ± 1.2	0.89
Amplitude prox [mV]	5.7 ± 2.3	10.2 ± 1.2	<*0.05*	4.5 ± 1.1	7.6 ± 1.3	<*0.05*
Amplitude mid [mV]	6.3 ± 2.3	12.4 ± 2.3	<*0.05*	5.6 ± 1.6	8.8 ± 1.6	<*0.05*
Amplitude dist [mV]	7.7 ± 1.5	14.1 ± 2.5	<*0.05*	7.4 ± 1.2	11.0 ± 1.4	<*0.05*
Duration prox [ms]	9.2 ± 2.7	4.7 ± 0.5	<*0.05*	11.2 ± 2.6	9.9 ± 2.1	0.2
Duration mid [ms]	9.1 ± 2.1	4.9 ± 0.6	<*0.05*	10.7 ± 2.0	9.7 ± 1.6	0.19
Duration dist [ms]	8.7 ± 2.5	4.2 ± 0.7	<*0.05*	9.9 ± 2.0	9.3 ± 1.9	0.45

Statistically significant differences are indicated in italics. THRT thyroid hormone replacement therapy

The BAER examination was performed in 14 patients in group A, including five dogs with vestibular signs. Dogs No. 2, 4, 14, 16 and 18, manifesting vestibular dysfunction, had increased peak I, II, III and V latencies with a normal I–V IPL and decreased amplitudes of wave I and II on the affected side when compared to group B (Table 4). The BAER recordings in 7/9 hypothyroid dogs without clinical vestibular dysfunction did not differ significantly from the control group regarding the analyzed parameters. The remaining two dogs (No. 7 and 13) had bilaterally increased peak V latencies and increased I–V IPL latencies. Dog No. 7 had unilaterally decreased amplitudes in the entire waveform (Table 4).

**Table 4:** Comparison of the BAER results between the control group and hypothyroid dogs with and without vestibular signs before and after initiating treatment with levothyroxine

	Latency [ms]				Interpeak latency	Amplitude [µV]			
	I	II	III	V	I–V	I	II	III	V
Group B (n = 10)									
Range	1.9–2.22	2.72–3.06	3.38–3.76	4.32–4.79	2.42–2.57	2.5–3.2	1.8–2.3	0.8–1.2	1.6–2.1
Mean	2.1	2.93	3.55	4.61	2.52	2.9	2.1	1.1	1.9
Group A not vestibular^a^ (n = 7)									
Range	2.0–2.26	2.88–3.07	3.35–3.74	4.4–4.8	2.4–2.6	2.6–3.1	1.7–2.4	0.6–1.1	1.5–1.9
Mean	2.15	2.99	3.48	4.68	2.54	2.9	2.1	0.8	1.8
Group A vestibular (n = 5) BT									
Ipsilateral									
Range	*2.45*–*2.58*	*3.15*–*3.27*	*3.84*–*4.0*	*4.99*–*5.3*	2.54–2.68	*0.9*–*1.8*	*1.1*–*1.5*	0.6–0.9	1.4–1.8
Mean	*2.47*	*3.18*	*3.91*	*5.13*	2.59	*1.5*	*1.3*	0.7	1.7
Contralateral									
Range	2.2–2.3	2.85–3.05	3.44–3.8	4.48–4.82	2.3–2.62	2.3–2.9	1.7–2.2	0.6–1.0	1.5–2.0
Mean	2.24	2.94	3.63	4.72	2.53	2.7	2.0	0.8	1.8
Group A vestibular (n = 5) AT									
Ipsilateral									
Range	2.36–2.58	3.15–3.23	3.85–3.9	4.9–5.13	2.5–257	1.4–2.2	1.5–2.0	0.7–10	1.4–1.9
Mean	2.51	3.18	3.89	5.09	2.55	1.8	1.7	0.8	1.7
Contralateral									
Range	2.2–2.44	2.84–3.05	3.41–3.83	4.46–4.9	2.23–2.52	2.4–2.8	1.7–2.1	0.6–1.0	1.5–2.1
Mean	2.29	2.92	3.66	4.75	2.45	2.6	1.9	0.8	1.8
Dog No. 7 BT									
L	2.22	2.92	3.81	*5.14*	*2.92*	2.3	1.7	0.7	1.7
R	2.21	3.03	3.83	*5.25*	*3.04*	*1.5*	*1.1*	*0.3*	*1.1*
Dog No. 7 AT									
L	2.2	2.91	3.77	4.81	2.61	2.4	1.7	0.8	1.7
R	2.21	3.04	3.8	4.83	2.62	1.7	1.4	0.6	1.5
Dog No. 13 BT									
L	2.05	3.09	3,76	*5.28*	*3.23*	2.5	2.1	1.1	2.0
R	2.08	3.1	3.79	*5.35*	*3.27*	2.4	2.2	1.0	1.8
Dog No. 13 AT									
L	2.04	2.81	3.7	4.59	2.54	2.5	2.3	1.1	1.7
R	2.05	2.89	3.75	4.62	2.58	2.5	2.0	0.9	1.9

Significant differences are indicated in italics. ^a^excluding cases No. 7 and 13. *BT* before treatment, *AT* after treatment [2 months], *L* left ear, *R* right ear

**Fig. 1:**
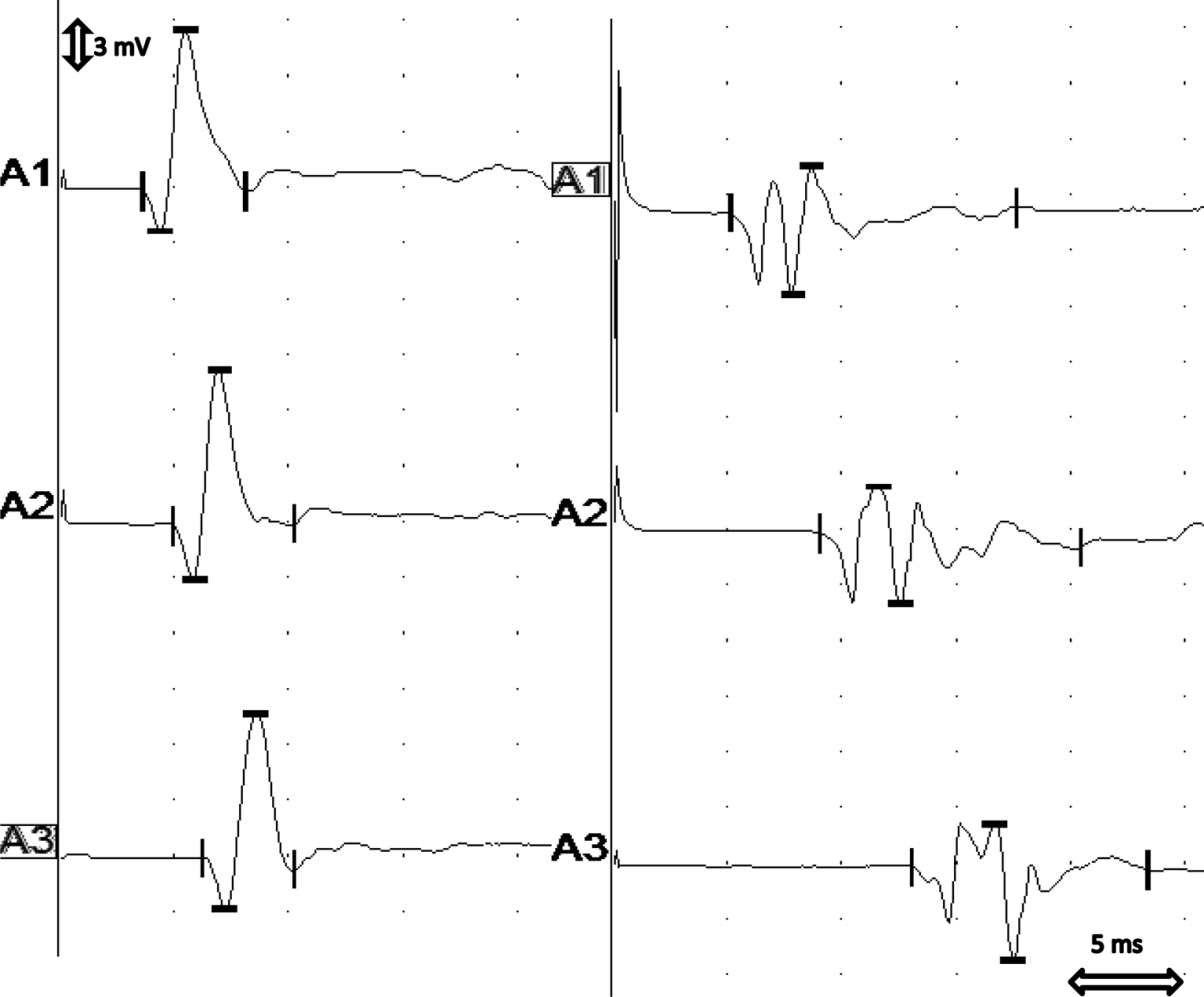
The comparison of nerve conduction studies in dogs from group A and B. The recording on the left was obtained from an 8-year-old mixed-breed dog from group B. The MNCV was 61–64 m/s and it was considered normal. The recording on the right was obtained from a 7-year-old Golden Retriever with suspected primary hypothyroidism. The MNCV was decreased to 30–31 m/s. *Decreased* CMAP amplitudes and *increased* CMAP duration (temporal dispersion) at all stimulation points were observed (*distal* A1, *middle* A2, *proximal* A3)

The results of the clinical, neurological and laboratory investigations in group B (control group) were within normal limits. The EMG examination of the control limbs showed electrically silent muscles with normal insertional activity. MNCV at the proximal–distal stimulation ranged from 62 to 71 m/s and from 57 to 64 m/s at the middle-distal stimulation site. The comparison of nerve conduction studies in dogs from group A and B is shown on Fig. 1. F wave latency values did not exceed expected latency values by more than 2 ms. The results of the BAER are presented in Table 4.

### Clinical and electrodiagnosting findings after initiation of levothyroxine supplementation

In group A, the first follow-up clinicopathological examination was performed 1 month after starting the levothyroxine treatment. At that time, all the studied dogs were more alert and active. The neurological evaluation revealed improvement in motor function by at least one Grade in 19/24 dogs. Dogs that showed no improvement after 1 month of the therapy and had a decreased tT4 level (No. 4 and No. 8) were administered a double levothyroxine dose (40 µg/kg BID). Dogs No 1, 5 and 9 had a tT4 level at a normal–low range. Because those dogs did not show a noticeable neurological improvement, levothyroxine supplementation was increased to 30 µg/kg BID. Dog No. 19 had a mildly increased serum tT4 concentration. Therefore, the levothyroxine dosage was decreased to 15 µg/kg BID.

Two months after initiating THRT, the results of the follow-up thyroid function tests and clinical examination were within normal limits in all 24 dogs. All overweight dogs lost weight. However, eight of them still exceeded recommended body mass. The majority of the vestibular signs resolved. Three dogs had a mild, persistent head tilt (No. 2, 16, 18). A full recovery of the motor function (Grade “0") was achieved in 13/24 dogs (No. 12–24). Eleven dogs that were initially scored as Grade “3" in 5 cases and Grade “2" in 6 cases (No. 1–11 in Table 1) showed an improvement to Grade “1" (ambulatory tetraparesis). This group was subjected to electrodiagnostic reexamination and named A1.

The EMG examination showed decreased pathological activity in all dogs. This improvement was from Grade “4" to “3" in 2/11 dogs, from Grade “4" to “2" in 2/11 dogs, from Grade “3" to “2" in 4/11 individuals and from Grade “3” to “1” in 3/11 dogs, as shown in Table 1. The analysis of the MNCV recordings showed statistically significant improvement of the proximal, middle and distal CMAP amplitudes and the proximal–distal conduction velocity when compared to the results before treatment (Table 3). The middle–distal conduction velocity, CMAP latencies and durations did not differ significantly before and after starting treatment in group A1 (Table 3). The F-wave was reexamined in the eight dogs that had a prolonged F-wave latency at the first examination (No. 1–6 and 10, 11). After a 2 month THRT therapy, there was no significant improvement in the F-wave latency in 5/8 dogs. The F-wave latency reached the normal value in 2/8 dogs. In one individual (dog No. 6), the latency shortened markedly, but the value still exceeded the expected F-wave latency by >2 ms (Table 1).

The BAER examination was repeated in five individuals with vestibular signs (dogs No. 2, 4, 14, 16 18) and two other dogs with an abnormal BAER recording during the first examination (No. 7 and 13) after 2 months of THRT. The second test revealed persistent prolongation of peak I, II, III and V latencies and a decreased wave I amplitude on the affected side in all dogs manifesting vestibular signs. The amplitude of wave II increased significantly in this group after replacement therapy (Table 4). In dogs without vestibular signs (No. 7 and 13), the peak V latencies and I–V IPL reached normal values after initiating therapy. Amplitudes in Dog No. 7 that were decreased prior to therapy increased markedly when measured at the second time point. However, they were lower than those obtained from the contralateral ear (Table 4).

## Discussion

### Diagnosis of polyneuropathy in hypothyroidism

Insufficient secretion of thyroid hormones is considered as a possible cause of polyneuropathy in 2–4 % of human patients [23]. Neurological abnormalities have been described in 6–29 % of dogs with naturally occurring hypothyroidism [1]. Peripheral neuropathy is the best documented neurologic manifestation of this disease in canine patients [8, 18]. However, a definitive diagnosis of canine primary hypothyroidism is complex [2]. The serum cTSH concentration has a high specificity for the diagnosis of hypothyroidism in dogs (more than 90 %) when the baseline serum T4 or fT4 concentration is concurrently low [24]. However, 20–40 % of dogs with primary hypothyroidism have normal cTSH levels, which gives the test low sensitivity [2]. A similar percentage of dogs with normal cTSH levels and decreased T4 and fT4 was present in our study (37.5 %). Caution should be taken in diagnosing hypothyroid neuropathy based only on a concurrent presence of low thyroid hormone levels [9]. Therefore, the presence of typical, extraneural clinical signs and accompanying hypercholesterolemia was required in this study to strengthen the suspicion of primary hypothyroidism in cases with normal cTSH. Despite the fact that a large number of additional tests was performed in order to rule out most systemic diseases, the nonthyroidal illness syndrome cannot be entirely excluded. Furthermore, we cannot exclude the possibility that symptoms in some dogs with acquired polyneuropathies of non-thyroidal etiology may resolve over a 2-month period. Hence, chronic inflammatory demyelinating polyneuropathy and distal denervating disease [9] may have been unintentionally included in the study.

The results indicate both a demyelinating and axonal pattern of polyneuropathy in dogs with a thyroid hormone deficiency. It is usually difficult to unequivocally distinguish between primary axonal and primary demyelinating polyneuropathy (i.e., neuropathy with extensive demyelination is often accompanied by slight axonal degeneration) [21]. Hypothyroid human patients have features of both axono and myelinopathy with a predominance (52–57 %) of demyelinating abnormalities in nerve conduction studies [11, 23, 25]. A prolonged CMAP and F-wave latency, a decrease in the nerve conduction velocity and temporal dispersion indicate demyelinating changes in our patients. Decreased CMAP amplitudes and EMG findings, which were also found in our study, stand for axonal involvement [26]. However, they may also indicate myopathy, especially in cases with elevated aspartate aminotransferase and creatine kinase activity [27]. In a study performed by Rossmeisl [27], a subclinical myopathy was the only electrodiagnostic finding in chronically hypothyroid dogs. No evidence of polyneuropathy, either clinical, or electrodiagnostic, was found in nine dogs with experimentally induced hypothyroidism. It has been suggested that immunological dysregulation contributes to peripheral nerve impairment in naturally-occurring hypothyroidism [27].

### Electrodiagnostic exam during the course of levothyroxine treatment

After initiation of levothyroxine supplementation, a range of electrophysiological findings have been described in human patients. According to Kececi et al. [25], polyneuropathy in hypothyroid patients can be reversed within 3 months using THRT. Significant improvement in MNCV, motor distal latency and amplitude values were reported. Nemni et al. [28] described complete recovery in the clinical status and a significant improvement in motor and sensory NCV, distal latency and amplitude in two patients following a 3 months replacement therapy. A similar improvement was observed after 6 months of supplementation in the remaining two patients. On the other hand, other authors demonstrated that nerve conduction studies did not show significant improvement, even after long-term replacement therapy [11, 23]. The reports regarding electrodiagnostic studies after replacement therapy in veterinary medicine are very limited. Budsberg et al. [15] noted a complete resolution of pathological EMG activity in three out of four dogs and partial improvement in the remaining dog after 2 months of levothyroxine supplementation. Similarly, Jaggy et al. [8] reported a complete improvement of EMG activity and MNCV variables in one hypothyroid dog with LMN signs 2 months after initiating replacement therapy. Our study showed that regardless of the clinical outcome, only some electrodiagnostic variables (CMAP amplitudes, proximal–distal MNCV and EMG) improved significantly after 2 months of replacement therapy in group A1.

### Hypothyroidism and BAER

Thyroid hormone deficiency has also been associated with auditory and vestibular system dysfunction in human [29] and canine patients [6, 8]. Vestibular signs may be the only clinical manifestation of concurrent, underlying polyneuropathy [8]. Vestibulocochlear nerve impairment can be associated with or occur independently of peripheral neuropathy [9]. All dogs in our study were presented with generalized polyneuropathy and 21 % of them manifested peripheral vestibular signs before treatment. The BAER findings, including ipsilaterally delayed peak latencies with a normal interpeak latency and markedly decreased wave I amplitude, which did not improve after treatment, are suggestive of vestibulocochlear neuropathy. The unilateral, isolated cranial nerve deficit may possibly be associated with a myxedematous compression of the nerve (in this case vestibulocochlear) as it exits through the foramen of the cranium [9]. The BAER examination in hypothyroid dogs without vestibular manifestation was within normal limits in 78 % of cases. The remaining individuals had bilaterally prolonged peak V latency and the I–V interpeak latency, indicating central conduction dysfunction. Jaggy et al. [8] reported that two hypothyroid dogs presenting with generalized LMN dysfunction had concurrent BAER abnormalities (bilaterally prolonged latencies or lack of response) without presenting vestibular signs. Thornton and Jarvis [29] reported a significant reduction in the amplitudes of waves III and V and an increase in the I–V interpeak latency (iPL) in the BAER in hypothyroid human patients. They stated that those abnormalities may be explained by the low body temperature of those individuals rather than retrocochlear involvement. In our study, the body temperature of the patients was within normal limits. Hence, we cannot concur with this explanation. However, the thyroid hormone is known to control protein synthesis and myelin production in the central auditory pathway [30]. In addition, T4 also acts as a neurotransmitter in the central nervous system [30]. This may be an explanation for central conduction dysfunction that was detected using the BAER in our patients. It has been suggested that BAER abnormalities and coexistent polyneuropathy in the course of acquired hypothyroidism may also be explained by segmental demyelination [6].

A two-month levothyroxine treatment resulted in a significant clinical improvement of the peripheral vestibular signs in our patients. A very good clinical outcome in dogs with vestibular disease and hypothyroidism was also reported by others [6, 8]. However, a mild, persistent head tilt was observed within 2 months of THRT in 77 % of the canine patients reported by Jaggy et al. [8] and 60 % of the dogs in our study.

Based on the BAER reexamination, we found that only partial improvement was achieved 2 months after the treatment initiation. In other veterinary reports, the complete resolution of BAER abnormalities was achieved after 6 weeks of treatment in one dog [6], and 3–5 months in two dogs [8].

## Conclusions

The results of the electrodiagnostic assessment in dogs with presumptive hypothyroidism and polyneuropathy showed features of both demyelination and axonal damage in the peripheral nerves. Most of the patients without vestibular signs showed neither peripheral nor central auditory pathway impairment, concurrent to the generalized neuropathy. The follow-up examination showed a very good clinical outcome and only partial improvement of the electrophysiological test results.

## Abbreviations

BAER: brainstem auditory evoked response
BID: twice daily
CMAP: compound muscle action potential
cTSH: canine thyroid stimulating hormone
EMG: electromyography
FPs: fibrillation potentials
fT4: free thyroxine
IPL: interpeak latency
MNCV: motor nerve conduction velocity
MS: millisecond
T4: total thyroxine
THRT: thyroid hormone replacement therapy
